# A Green Magnetite-Coated Waste Biomass for Simultaneous Removal of Multiple Toxic Metals from Water

**DOI:** 10.1021/acsomega.6c00870

**Published:** 2026-06-24

**Authors:** Tülin Deniz Çiftçi, Yağmur Deniz Çiftçi, Ela Güngör, Zeliha Ada İplikçi, Serap Yıldırım Metin

**Affiliations:** † 322429 Ege University, Faculty of Science, Department of Chemistry, Erzene, Bornova, İzmir 35100, Türkiye; ‡ İzmir Bahçeşehir College 50th Year Anatolian High School, Çiçekliköy, Bornova, İzmir 35040, Türkiye

## Abstract

The presence of multiple toxic metals in water poses a serious environmental and public health risk, yet most adsorption studies focus on single-component systems that do not adequately represent real contamination scenarios. In this study, a low-cost adsorbent was prepared by coating waste keratin-based biomass fibers with Fe_3_O_4_ nanoparticles and evaluated for the simultaneous removal of 14 toxic metal and metalloid ions from water. Batch adsorption experiments demonstrated removal efficiencies in the range of approximately 80–99% for different target ions under multielement conditions across a broad pH range. Multimodel kinetic and equilibrium behavior was observed depending on the element type, and adsorption evaluation was based on overall trends and consistency between experimental and calculated uptake values. Competitive adsorption among coexisting ions was also investigated. The applicability of the adsorbent was further confirmed using real drinking water and wastewater samples, yielding average recovery values of around 90%, indicating satisfactory performance in complex matrices. The results indicate that magnetite-coated waste biomass is a promising and sustainable adsorbent for multimetal water remediation under realistic conditions.

## Graphical abstract

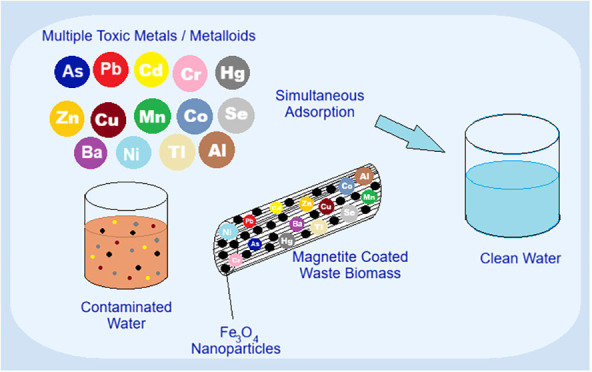

## 1 Introduction

Heavy metals and metalloids are natural substances naturally present in the environment.^1^ These compounds found in soil can enter water and air by natural and/or human means. As potable water resources are increasingly strained, it is essential to treat contaminated water and remove toxic compounds to ensure water safety.^2^ In terms of health effects, many are known to have carcinogenic effects and, like lead, have significant toxic health effects on children during pregnancy and early childhood.^3^ Due to these effects, organizations such as the World Health Organization^4^ and the Environmental Protection Agency^5^ recommend certain concentration values for toxic substances that can be found in water.

Numerous studies have investigated this topic. Although various methods such as precipitation, coagulation, reverse osmosis are used, adsorption stands out as a particularly effective method due to its superior properties.^6^

A large number of adsorbents for the treatment of different species, obtained by different methods, have been reported. Waste-derived materials have been widely investigated as adsorbents.For example, waste materials such as discarded cigarette butts,^7^ citrus waste,^8^ spent coffee^9^ have been effectively used as adsorbents for heavy metal removal. For the same purpose, green and eco-friendly^10–13^ materials have been frequently used for heavy metal removal. A natural byproduct material, pressed black cumin cake^14^ was used for the removal of copper. The capacity of the material for the adsorption of copper was 106.38 mg/g. Gokmen et al. used eco-friendly Polyacrylic acid based (PAA) hydrogel for the adsorption of Cd­(II), Ni­(II), Cu­(II) and Fe­(III).^15^ They obtained the highest adsorption efficiency in the adsorption of Cd­(II) ions. Vargas-Solano et al. used Opuntia ficus-indica (OFI) mucilage for the removal of heavy metals in the Yautepec River.^16^ The stem bark of Ximenia americana L. has been demonstrated to act as an effective biosorbent for Pb­(II) removal from aqueous effluents, achieving significant lead uptake and confirming the capability of plant-derived waste biomaterials for heavy metal remediation.^17^ In the study, they reported that different treatment efficiencies were obtained depending on the optimal pH values and the heavy metal concentrations in the river (96% Fe, 91% Mn, 60% Cr, 70% As, and less than 40% of Cd, Ni and Pb).

Recently, advanced sorbent systems based on synthetic coordination networks have demonstrated excellent performance for the removal of multiple metal ions from aqueous systems.^18^ However, many of these materials require specifically designed chemical structures or synthesis steps. In contrast, the present study explores a waste-derived keratin-based magnetic adsorbent obtained from human hair, providing a simple and sustainable alternative for multielement removal from real water matrices.

In recent years, nanomaterials have been frequently used due to their high capacity, easy separation from the solution medium and high water treatment efficiency.^19^ The most common among these nano materials is magnetite compound. Magnetite is frequently used for arsenic,^20^ lead,^21^ cadmium,^22^ chromium,^23^ dye^24^ removal. It has also been used for treatment purposes when coated on support materials such as nutshell,^25^ sand,^26,27^ biochar.^28^

Although various techniques are developed for synthesizing nanoparticles, researchers are currently focused on developing green, biocompatible, and cost-effective methods to synthesize these materials. However, studies are often focused on the removal of only one or two toxic substance. For this reason, different materials will be prepared for each toxic substance in waters polluted by heavy metals, and thus the costs will increase considerably.

Despite extensive research on adsorbent materials for metal removal, most studies remain limited to single- or binary-metal systems, which fail to capture the complexity of real contaminated waters containing multiple competing ions. Moreover, the practical applicability of many reported materials is constrained by high cost, difficult recovery, or limited performance in real water matrices. To address these limitations, this study investigates a magnetite-coated waste biomass adsorbent for the simultaneous removal of 14 toxic metals and metalloids. The adsorption behavior under multielement conditions, competitive effects, and performance in real drinking water and wastewater samples were systematically evaluated. This work provides an application-oriented assessment of a sustainable adsorbent material for realistic water remediation scenarios.

## 2 Materials and Methods

All batch adsorption experiments were performed in triplicate, and the results are reported as mean ± standard deviation (SD). Linearized isotherm and kinetic models were initially applied as comparative tools for preliminary evaluation across multiple contaminants. Due to the complexity of the multicomponent system involving 14 simultaneously competing elements, these models were used for descriptive comparison rather than absolute mechanistic determination. To further validate the model interpretation, representative nonlinear isotherm and kinetic analyses were additionally performed for selected elements and are presented in the Supporting Information (Figures S2 and S3).

### 2.1 Reagents

All the reagents were of analytical-reagent grade and ultrapure water was used throughout. FeCl_2_·4H_2_O, FeCl_3_·6H_2_O, NaOH and acetone (Merck or Sigma) were used for the preparation of the adsorbents. QCS-27 (ChemLab) multielement solution (100 mg/L) was used for the adsorption experiments.

### 2.2 Apparatus and Instrument

Analysis of the elements were performed by ICP-MS (Agilent/7900 Inductively Coupled Plasma-Mass Spectrometer) instrument. SEM -EDX (Jeol/JSM-6610), pH meter (Mettler Toledo/FG2) and orbital shaker (Biosan/OS-10) were also used for the analysis.

Quadrupole ICP-MS measurements were carried out using an Agilent 7900 instrument operated in standard (General Purpose plasma) and He collision modes via MassHunter software. Prior to analysis, the instrument was optimized with a multielement tuning solution (Ce, Co, Li, Tl, Y; Agilent Technologies) and stabilized for 30 min using 2% (v/v) HNO_3_.

External calibration was performed using multielement standards (Redoks Lab) prepared in 2% (v/v) HNO_3_. An internal standard mixture (Bi, Ge, In, Li, Lu, Rh, Sc, Tb; Agilent Technologies) was used to correct for instrumental drift and matrix effects.

Instrumental conditions were set as follows: RF power 1300 W, cell gas flow rate 4.3 mL/min, sample uptake rate 0.5 mL/min, and peristaltic pump speed 20 rpm. The monitored isotopes were ^27^Al, ^52^Cr, ^55^ Mn, ^56^Fe, ^60^Ni, ^78^Se, ^111^Cd, ^121^Sb, ^137^Ba, and ^207^Pb. Calibration was performed using external multielement standards and analytical quality was verified through internal standard correction and blank measurements.

The detailed calibration curves (including regression equations and coefficients of determination R^2^ values), as well as the limits of detection (LOD) and limits of quantification (LOQ) for all analytes, are provided in the Supporting Information (Table S1 and Figure S1).

### 2.3 Preparation of the Adsorptive Materials

Human hair was selected as the keratin source due to its well-defined cuticle–cortex morphology and high availability as urban waste. The overlapping cuticle scales provide a textured surface that facilitates iron ion adsorption and subsequent in situ formation of Fe_3_O_4_ nanoparticles. Compared to other keratinous wastes such as feathers (β-keratin) or wool (α-keratin with surface lipids), human hair offers a relatively uniform fiber geometry and accessible surface functional groups, supporting homogeneous magnetite deposition.

The keratin-rich waste biomass fibers were derived from discarded human hair collected from local barbershops, washed, dried, and processed prior to use. The photos of all the stages are shown in Figure 1.

**Figure 1:**
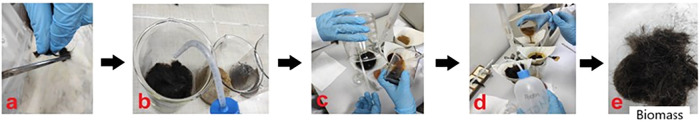
Preparation of the support materials. (a) Shortening the length, (b) washing with ultrapure water, (c) transferring to filter paper, (d) washing with acetone and filtering through filter paper, (e) cleaned keratin-based biomass.

FeCl_2_·4H_2_O and FeCl_3_·6H_2_O solids were used for the preparation of 0.25 M Fe­(II): 0.50 M Fe­(III) solutions, respectively. Thirty mL of iron solution was added to 1.0 g of biomass fibers and shaken in an orbital shaker at 300 rpm for 24 h. The supernatant solutions were decanted and 1.0 M NaOH solution (∼70 °C) was added dropwise. Magnetite was formed (eq 1) from iron ions held in the keratin-based biomass fiber cuticles.The adsorbents were shaken with this solution for 24 h. The supernatant solution was decanted and washed with ultrapure water and acetone. The resulting adsorbents were dried at room temperature (25 °C). The stages are shown in Figure 2.

1 $${\mathrm{FeCl}}_{2}+2{\mathrm{FeCl}}_{3}+8\mathrm{NaOH}\to {\mathrm{Fe}}_{3}O_{4}+8\mathrm{NaCl}+4H_{2}O$$

**Figure 2:**
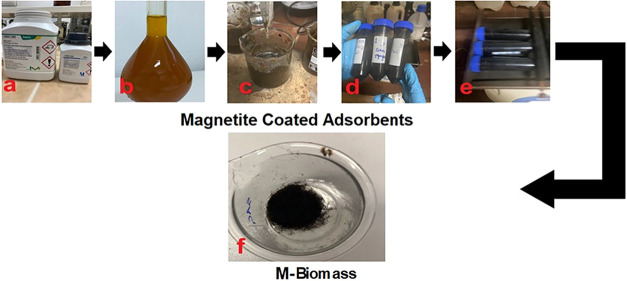
Supporting magnetite on the support materials. (a) FeCl_3_·6H_2_O and FeCl_2_·4H_2_O solids used to form magnetite, (b) solution prepared from solids, (c) photographs after adding NaOH, (d) mixtures taken into falcon tubes before shaking, (e) shaking in orbital shaker, (f) M-biomass, magnetite coated biomass.

## 3 Results

### 3.1 Comparison of the Adsorption Efficiencies

A 1.0 mg/L metal solution was prepared from a stock solution (100 mg/L). 0.1 g of adsorbents were weighed and 30 mL of metal solution was added. After shaking for 24 h, the supernatant solutions were taken into clean falcon tubes and the metal concentrations were determined with the ICP-MS instrument. To determine whether magnetite coating was effective or not, the same adsorption study was also carried out with uncoated biomass materials. Removal efficiencies were calculated and are shown in Table 1.

**Table 1:** Removal Efficiencies of Magnetite Coated and Uncoated Biomass^a^

	coated adsorbent (R.E. %)	uncoated adsorbent (R.E. %)
aluminum	76.4 ± 3.1	12.590 ± 0.051
chromium	**77.8 ± 1.2**	**98.9 ± 2.5**
manganese	93.8 ± 7.5	2.110 ± 0.011
cobalt	**98.7 ± 4.2**	3.835 ± 0.015
nickel	**99.7 ± 8.4**	5.506 ± 0.024
copper	**93.9 ± 5.6**	77.6 ± 1.1
zinc	**95.9 ± 1.8**	7.000 ± 0.015
arsenic	**72.71 ± 0.85**	64.65 ± 0.33
selenium	**55.98 ± 0.48**	25.630 ± 0.072
silver	**87.5 ± 1.9**	12.816 ± 0.056
cadmium	**99 ± 10**	26.30 ± 0.16
barium	91.3 ± 1.6	9.414 ± 0.045
thalium	69.86 ± 0.20	15.742 ± 0.063
lead	78.41 ± 0.41	**97.7 ± 4.0**

a C: 1.0 mg/L; pH:5.0; adsorbent: 0.1 g; *t*: 24 h, *n* = 3. R.E. is removal efficiency.

Magnetite coating generally improved the adsorption performance for most of the investigated metal ions, resulting in higher removal efficiencies compared to the uncoated biomass. However, for a limited number of elements such as Cr and Pb, comparable or slightly higher removal efficiencies were observed for the uncoated biomass. This behavior may be attributed to the inherent binding affinity of keratin functional groups toward certain metal ions and the complex competitive adsorption conditions in multielement systems. Overall, when all investigated species are considered collectively, magnetite coating enhances the adsorption capacity and broadens the range of effectively removed contaminants.

Additionally, it was determined that minerals such as Na, B, Si, K, which are necessary and beneficial for the human body, were not adsorbed quantitatively, while Mg and Ca were adsorbed at most by 25%. Thus, we have prepared a material that adsorbs toxic elements and does not have a major negative impact on beneficial ones.

### 3.2 Surface Characterization of the Adsorbent

SEM (Scanning Electron Microscopy) and EDX (Energy Dispersion X-ray Spectrometry) analyzes were performed for the surface characterization of the magnetite coated biomass adsorbent. Au/Pd coating was applied to the adsorbent before analysis for the analysis of magnetite. SEM and EDX analysis results are shown in Figures 3 and 4, respectively.

**Figure 3:**
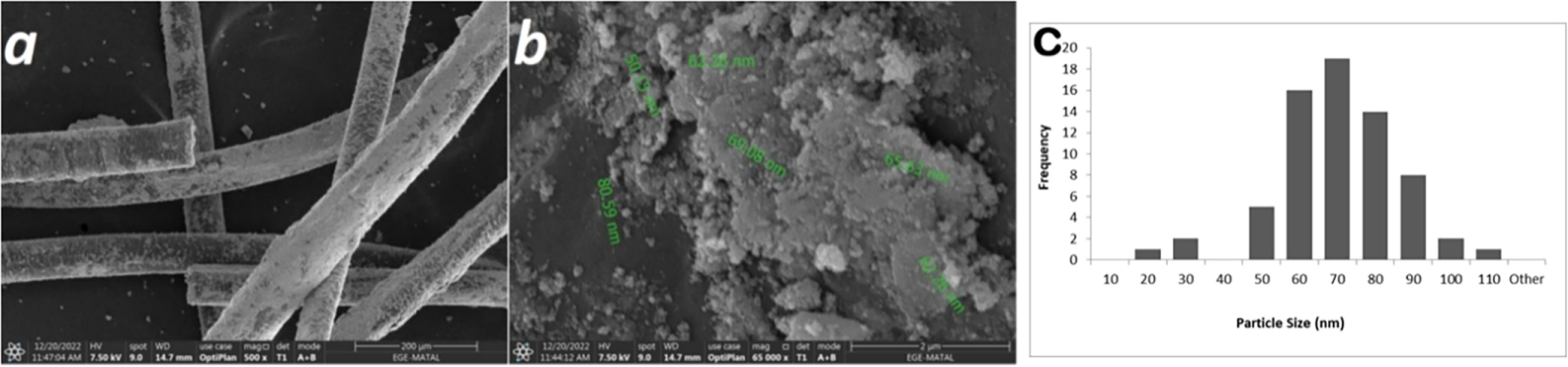
SEM images of magnetite coated biomass fibers adsorbent at (a) 500× and (b) 65,000× magnifications. (c) Particle size distribution histogram.

**Figure 4:**
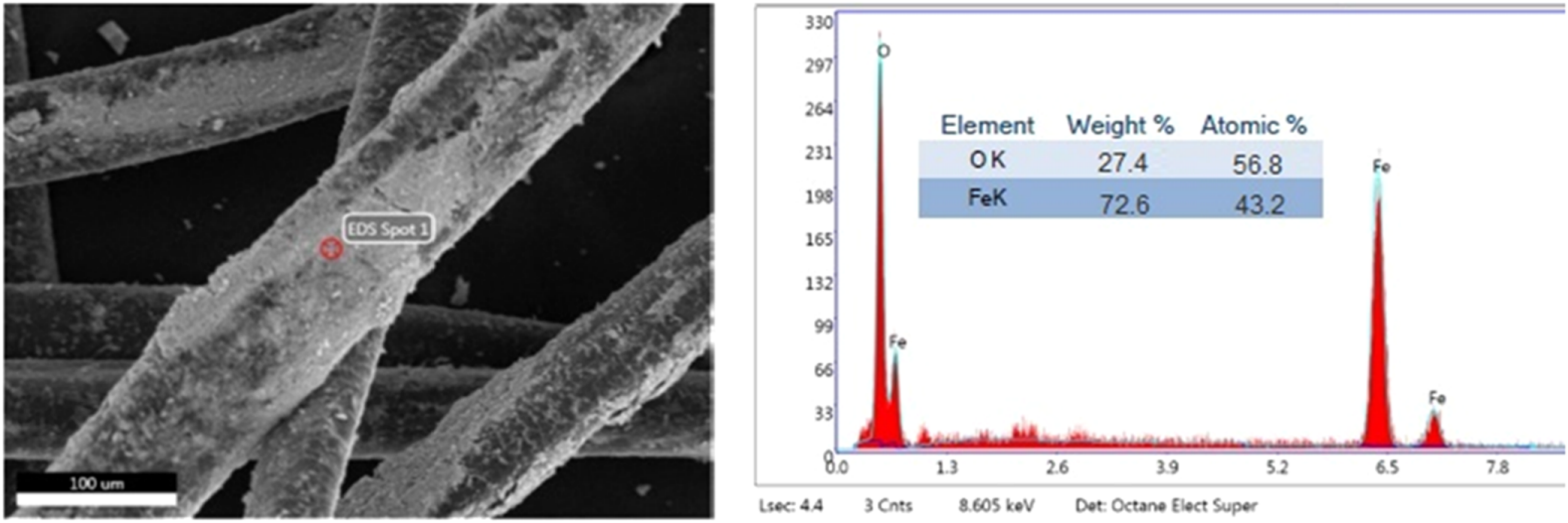
EDX analysis result of the adsorbent.

As shown in Figure 3a, magnetite particles were successfully deposited on the biomass fibers and within the cuticle structures of the hair matrix. The SEM image (Figure 3b) reveals the presence of nanoscale magnetite particles distributed on the surface. To quantitatively evaluate particle size, a statistical analysis was performed by measuring 72 randomly selected particles from the SEM micrograph using ImageJ software. The resulting particle size distribution is presented as a histogram in Figure 3c. The nanoparticles exhibit an average diameter of 63.5 ± 18.3 nm, with most particles predominantly distributed within the 50–90 nm range, confirming the formation of nanoscale magnetite on the biomass surface. The relatively uniform nanoscale dispersion of magnetite particles suggests successful nucleation and growth of Fe_3_O_4_ on the keratin matrix. Such nanoscale distribution is expected to enhance the available active surface area and facilitate metal ion adsorption during water treatment.

It has been confirmed that the magnetite structure formed on the adsorbent was successfully obtained (Figure 4). Considering the weight and atomic ratios, it was determined that the structure was compatible with the magnetite compound. The Fe_3_O_4_ compound theoretically contains 27.6% oxygen and 72.4% iron by weight. Similarly, the Fe_3_O_4_ compound contains 42.9% oxygen and 57.1% iron element atomically.

### 3.3 Determination of the Surface pH of the Adsorbent

Determination of surface pH is very important in order to determine the relationship between adsorbent and pollutants in the adsorption method. The surface pH (pH_p_zc) of the adsorbent was determined using the pH drift method. Briefly, 0.05 g of adsorbent was added to 25 mL of 0.1 M NaNO_3_ solution to maintain constant ionic strength. The initial pH of the solution was adjusted in the range of 3–12 using 0.1 M HNO_3_ or 0.1 M NaOH solutions. The suspensions were shaken at 300 rpm for 24 h to ensure equilibrium. After equilibration, the final pH was measured, and ΔpH (pH_final_ – pH_initial_) was plotted against the initial pH. The pH_pzc_ was determined as the point where ΔpH = 0, corresponding to the intersection of the curves (Figure 5). The pH 10.1 point, where the graph crosses the *x*-axis, was determined as the surface pH.

**Figure 5:**
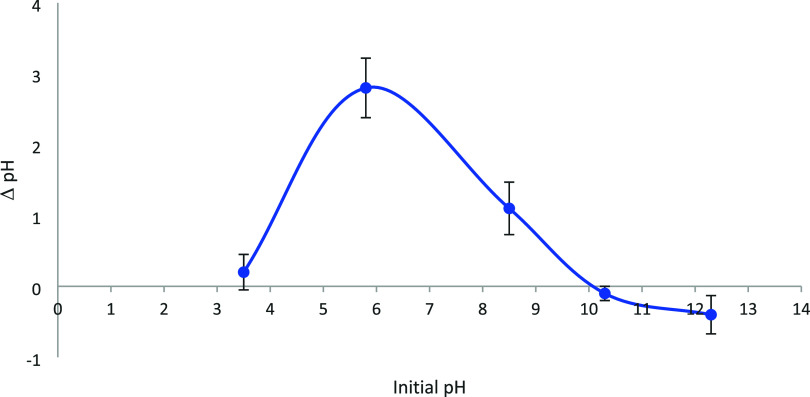
ΔpH values versus initial pH (pH:3–12; adsorbent: 0.05 g; *t*: 24 h).

2 ΔpH=equilibrium⁢pH−initial⁢pH

The relatively high pH_p_zc value (10.1) indicates that the adsorbent surface remains positively charged under most environmentally relevant pH conditions (pH < 10.1). This suggests that the surface charge of the material is strongly influenced by protonation–deprotonation equilibria of functional groups on keratin and surface hydroxyl groups on Fe_3_O_4_. Above the pH_p_zc value, the surface gradually becomes negatively charged due to deprotonation of these groups.

### 3.4 Effect of pH

0.05 g of adsorbent was added to 500 μg/L metal solutions (prepared at different pHs) and shaken for 24 h for the determination of the optimum adsorption pH. The concentrations of unadsorbed metals were determined by ICP-MS instrument. pH-dependent removal efficiencies were calculated. The results are shown in Figure 6.

**Figure 6:**
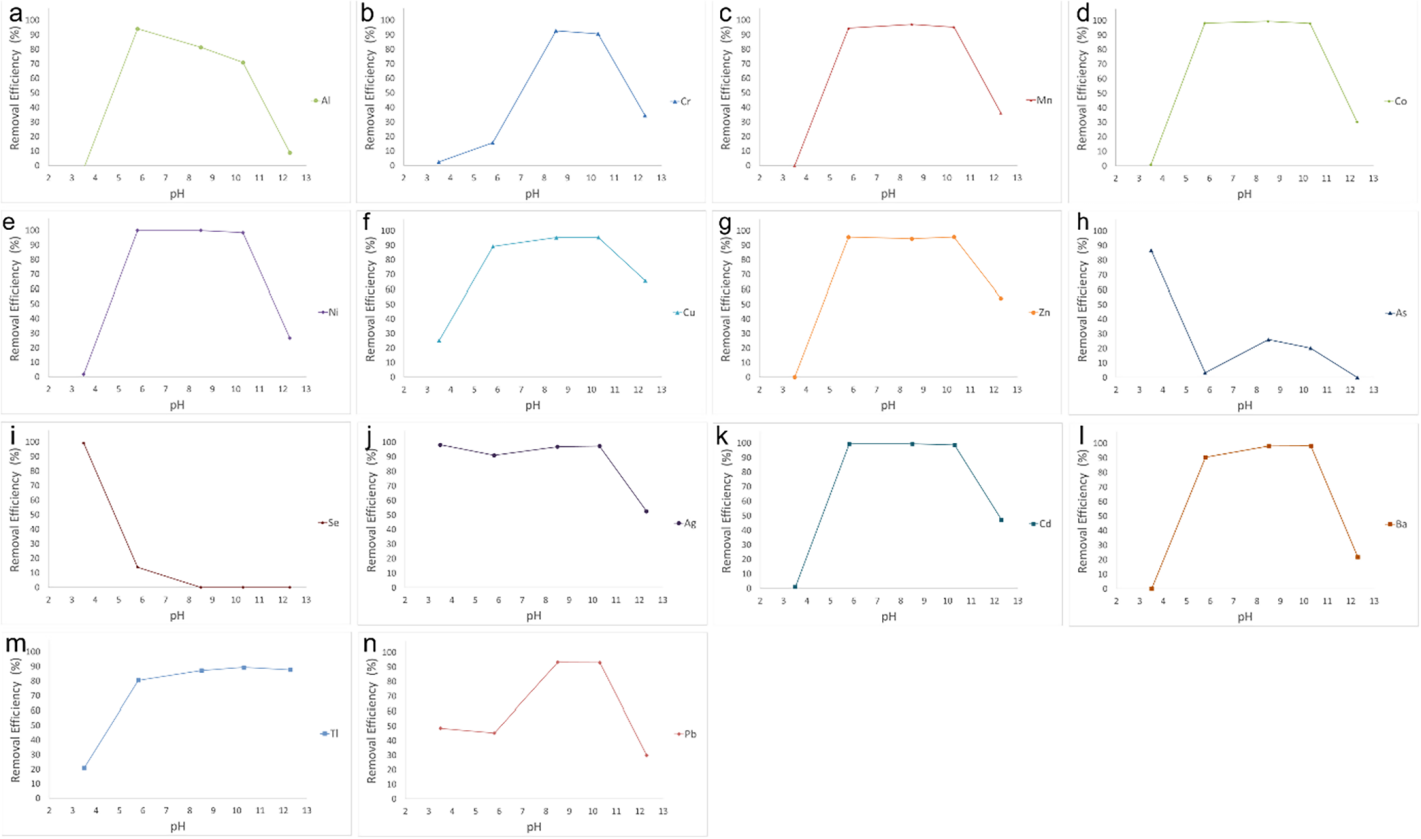
Removal efficiencies of magnetite coated biomass adsorbent at different pH values. (a) Al, (b) Cr, (c) Mn, (d) Co, (e) Ni, (f) Cu, (g) Zn, (h) As, (i) Se, (j) Ag, (k) Cd, (l) Ba, (m) Tl, (n) Pb (C: 0.5 mg/L; pH: 3–12; adsorbent: 0.05 g; *t*: 24 h).

Different optimum pH values were determined for each of the metals as can be seen in Figure 6. Optimum pH values are also shown in Table 2.

**Table 2:** Optimum pH Values and the Removal Efficiencies for the Adsorption of Elements (*n* = 3)

element	optimum pH	removal efficiency (%)
aluminum	5.8	94.13 ± 0.98
chromium	8.5	92.7 ± 1.7
manganese	8.5	97.1 ± 2.2
cobalt	8.5	99.4 ± 2.1
nickel	5.8–8.5	>99.9 ± 1.0
copper	8.5–10.3	95.4 ± 2.0
zinc	5.8–10.3	95.7 ± 1.4
arsenic	3.5	86.807 ± 0.050
selenium	3.5	99.3 ± 2.1
silver	3.5–10.3	>97.0 ± 1.4
cadmium	5.8–10.3	>99.0 ± 0.20
barium	5.8–8.5	98.3 ± 1.8
thalium	8.5 – 12.3	>87.2 ± 2.3
lead	8.5–10.3	>93.0 ± 1.5

As shown in Table 2, treatment was more effective in acidic media for some elements, whereas most elements exhibited higher removal efficiencies in basic media. Although some metal ions may precipitate as insoluble hydroxides at alkaline pH, the controlled experimental conditions and literature solubility considerations suggest that precipitation is expected to play a limited role under the studied conditions, and the observed removal is primarily attributed to adsorption onto the magnetite-coated biomass.

Although the point of zero charge (pH_pzc_ = 10.1) indicates that the adsorbent surface remains positively charged up to alkaline conditions, the optimum adsorption for most metal cations was observed at pH values below this point, typically around pH 8.5. This apparent difference can be explained by the combined effects of surface charge evolution, metal speciation, and functional group chemistry. At pH values near 8.5, partial deprotonation of keratin functional groups (−COOH and −NH_2_) together with the formation of reactive surface hydroxyl groups on Fe_3_O_4_ enhances metal–surface complexation and coordination interactions. In addition, many metal ions begin to form hydrolyzed species at this pH range, which generally exhibit higher affinity toward heterogeneous and functionalized surfaces. Therefore, adsorption is governed not only by electrostatic interactions but also by a synergistic combination of surface complexation, ligand exchange, and metal hydrolysis effects.

### 3.5 Effect of the Adsorbent Dose

Twenty-five mL of 1.0 mg/L metal solutions were added to adsorbents of different weights and shaken for 24 h in order to determine the amount of adsorbent to be used in adsorption experiments. Metal concentrations remaining in the solution were determined. The adsorbent dose versus the removal efficiency graphs were drawn and are shown in Figure 7.

**Figure 7:**
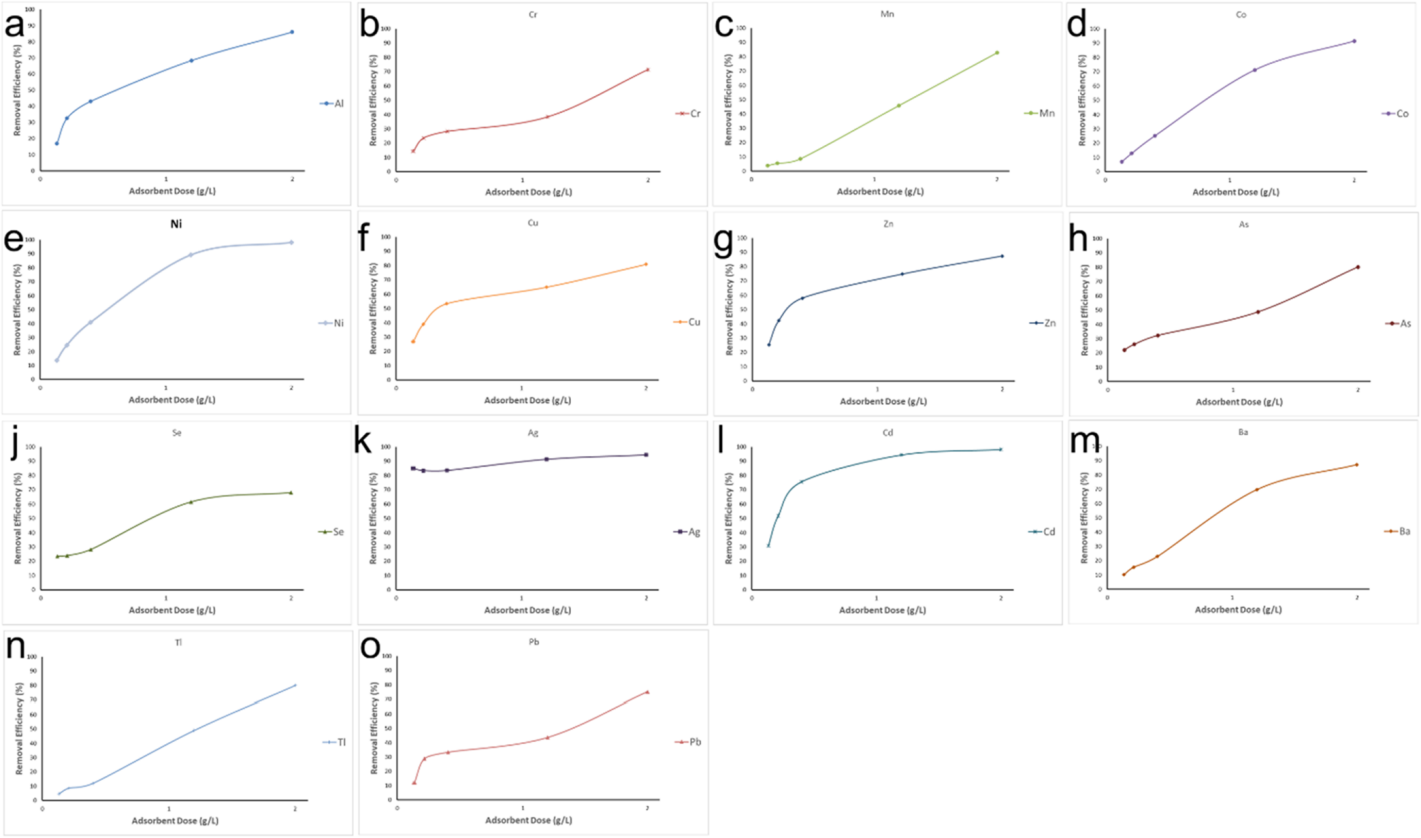
Removal efficiencies (%) versus the adsorbent doses. (a) Al, (b) Cr, (c) Mn, (d) Co, (e) Ni, (f) Cu, (g) Zn, (h) As, (j) Se, (k) Ag, (l) Cd, (m) Ba, (n) Tl, (o) Pb (C: 1.0 mg/L; pH:7.0; *t*: 24 h).

Metals that showed higher removal efficiency in the acidic region had low removal efficiency in this experiment (at pH 7). When the element that causes water pollution is determined, high removal efficiency can be achieved by working at the optimum pH value. The highest removal efficiencies were obtained for all elements at a 2 g/L adsorbent dose.

### 3.6 Adsorption Isotherms

Adsorption isotherms are used to determine the adsorbent capacity with parameters such as whether the adsorption is monolayer or multilayer, homogeneous or heterogeneous.

The Langmuir isotherm model^29^ explains that monolayer adsorption occurs on homogeneous surfaces. The linearized Langmuir isotherm equation (eq 3) is shown below.

3 $$\frac{C_{e}}{q_{e}}=\frac{1}{bQ_{\max}}+\frac{C_{e}}{Q_{\max}}$$

where, *C*_e_ is the element concentration at equilibrium (μg/L), *q*_e_ (μg/g) is the amount of adsorbed elements per unit amount of adsorbent at equilibrium, *C*_0_ is the initial element concentration (μg/L), *Q*_max_ is the maximum adsorption capacity (μg/g), *b* is the Langmuir constant (L/μg).

The Freundlich isotherm model^30^ describes heterogeneous adsorption. The linearized form of the Freundlich isotherm is

4 $$\ln q_{e}=\ln K_{f}+\frac{1}{n}\ln C_{e}$$

where *K*_f_ is the Freundlich constant (μg/g), *n* is the Freundlich constant (unitless).

All equations, *R* ^2^ values, and isotherm constants of the adsorption isotherm graphs obtained from Langmuir and Freundlich isotherms are shown in Table 3.

**Table 3:** Equations of the Adsorption Isotherms Graphs, *R* ^2^ Values and Isotherm Constants

	Langmuir				Freundlich			
	equation	*R* ^2^	*b* (L/μg)	*Q*_max_ (μg/g)	equation	*R* ^2^	*n*	*K*_f_ (μg/g)
Al	*y* = 0.3995*x* + 0.2623	0.7668	1.52	2503	*y* = 0.6823*x* + 0.2218	**0.9428**	1.47	1666
Cr	*y* = 0.8797*x* + 0.5616	0.953	1.57	1136	*y* = 0.5578*x* – 0.1468	**0.9861**	1.79	713
Mn	*y* = 4.3635*x* – 0.5189	**0.9083**	8.41	229	*y* = −0.2859*x* – 0.5769	0.644	3.50	264
Co	*y* = 1.7356*x* – 0.0009	**0.9747**	1928	576	*y* = 0.0902*x* – 0.22	0.4922	11.1	603
Ni	*y* = 0.887*x* + 0.0348	**0.9898**	25.49	1127	*y* = 0.2116*x* + 0.0611	0.9763	4.73	1151
Cu	*y* = −0.3856*x* + 0.6123	0.3781	0.63	2593	*y* = 1.3257*x* + 0.501	**0.9076**	0.75	3170
Zn	*y* = 0.1342*x* + 0.2515	0.5089	0.53	7452	*y* = 0.894*x* + 0.463	**0.971**	1.12	2904
As	*y* = 0.5149*x* + 0.4095	0.8185	1.26	1942	*y* = 0.6538*x* + 0.0563	**0.9707**	1.53	1138
Se	*y* = −0.7468*x* + 1.1145	0.7154	0.67	1339	*y* = 1.5935*x* + 0.3425	**0.9385**	0.63	2200
Ag	*y* = −0.5844*x* + 0.1582	0.8086	3.69	1711	*y* = 1.7483*x* + 1.8093	**0.9139**	0.57	64461
Cd	*y* = 0.3571*x* + 0.0401	**0.9874**	8.91	2800	*y* = 0.4741*x* + 0.5078	0.9764	2.11	3220
Ba	*y* = 1.2513*x* + 0.1555	**0.9165**	8.05	799	*y* = 0.2403*x* – 0.137	0.7843	4.16	729
Tl	*y* = 4.6123*x* – 0.7022	**0.9157**	6.59	216	*y* = −0.3022*x* – 0.5705	0.5952	3.31	269
Pb	*y* = 1.2437*x* + 0.2999	**0.8381**	2.05	1100	*y* = 0.425*x* – 0.1556	0.7544	2.35	699

The adsorption isotherm behavior of manganese, cobalt, nickel, cadmium, barium, thallium and lead showed adsorption trends more consistent with Langmuir-type fitting, while aluminum, chromium, copper, zinc, arsenic, selenium and silver exhibited adsorption behavior that could be represented using the Freundlich model. These observations suggest that both relatively uniform and heterogeneous adsorption sites may coexist on the keratin–magnetite surface. Model interpretation was performed cautiously by considering agreement between calculated and experimental adsorption capacities together with overall adsorption trends, rather than relying solely on *R* ^2^ values.

The differences observed in isotherm behavior among the investigated elements can be attributed to their distinct chemical speciation and interaction mechanisms with the keratin–magnetite surface. Transition metal cations (e.g., Mn^2+^, Co^2+^, Ni^2+^, Cd^2+^, and Pb^2+^) can form coordination interactions with functional groups present in keratin, such as amino (−NH_2_), carboxyl (−COO^–^), and thiol (−SH) groups, as well as with surface hydroxyl groups of magnetite. Such interactions tend to favor localized binding sites and may lead to adsorption behavior consistent with the Langmuir model. In contrast, elements such as As and Se typically occur in aqueous solutions as oxyanions (e.g., arsenate or selenite species), which interact with the adsorbent surface mainly through electrostatic attraction and surface complexation mechanisms. These processes may occur on energetically heterogeneous sites, resulting in adsorption behavior better described by the Freundlich isotherm.

Furthermore, a clear distinction can be observed between metal ions and metalloids. Metal cations are more likely to interact with keratin functional groups (−NH_2_, −COOH, −SH) through coordination and complexation mechanisms, whereas metalloids such as arsenic and selenium, which are typically present as oxyanions, may interact via ligand exchange, hydrogen bonding, and surface complexation on magnetite sites. The coexistence of keratin functional groups and magnetite nanoparticles enables the adsorption of both cationic and anionic species through different but complementary mechanisms. This multimechanistic behavior is particularly advantageous for treating complex water matrices containing diverse contaminants and highlights the practical applicability of the developed adsorbent.

### 3.7 Kinetic Studies

Kinetic models are used to investigate the adsorption mechanism and rate control steps. For this purpose, a detailed kinetic study was carried out. 100 mL of 1000 μg/L element solutions were added onto 0.2 g of adsorbent and shaking was started. Sample solutions were taken from the supernatant solution at zeroth, 15th, 45th, 75th, 150th, 270th, 340th and 1440th minutes and analyzed.

Three kinetic models were applied to the experimental data of 14 elements. The adsorption kinetics of elements have been studied with Lagergren pseudo-first-order (PFO),^31^ pseudo-second-order (PSO)^32^ and Weber-Morris intraparticle diffusion^33^ models.

Kinetic model equations are given below:

Pseudo-first-order model

5 $$\log\left(q_{e}-q_{t}\right)=\log q_{e}-\frac{k_{1}}{2.303}t$$

Pseudo-second-order model

6 $$\frac{t}{q_{t}}=\frac{1}{h}+\frac{t}{q_{e}},\;h=k_{2}\cdot {q_{e}}^{2}$$

Weber Morris intraparticle diffusion model

7 $$q_{t}=k_{\mathrm{int}}t^{0.5}+I$$

Here, *q*_e_ and *q_t_* (μg/g) are the amount of elements adsorbed on the adsorbent at equilibrium and time *t*, respectively. *t*, time (min), *k*_1_ (1/min) is the rate constant of pseudo-first-order adsorption, *k*_2_ (g/μg·min) is the rate constant of pseudo-second order adsorption, *k*_int_ (μg/g·min^0.5^) is the rate constant of intraparticle diffusion.

The data obtained from kinetic experiments were applied to kinetic models and graphs were drawn. Graph equations, coefficients of determinations (*R* ^2^) and constants are shown in Tables 4 and 5.

**Table 4:** Parameters of Pseudo-First-Order and Pseudo-Second-Order Kinetic Models^a^

	pseudo first order				pseudo second order				
	equation	*R* ^2^	*k*_1_	*q*_e_	equation	*R* ^2^	*k*_2_	*q*_e_	*h*
Al	*y* = −0.0002*x* + 2.627	**0.990**	0.0005	424	*y* = 0.0037*x* + 0.7675	0.939	1.78 × 10^–5^	270	1.3
Cr	*y* = −0.00004*x* + 2.6897	**0.974**	0.0001	489	*y* = 0.0096*x* + 7.3421	0.649	1.26 × 10^–5^	104	0.1
Mn	*y* = −0.0002*x* + 2.0341	0.764	0.0005	108	*y* = 0.0022*x* + 0.0241	**1.000**	0.000201	455	41.5
Co	*y* = −0.0002*x* + 1.9297	0.575	0.0005	85	*y* = 0.0022*x* + 0.0133	**1.000**	0.937647	455	75.2
Ni	*y* = −0.0002*x* + 2.5581	0.901	0.0005	36	*y* = 0.0021*x* + 0.0062	**1.000**	7.11 × 10^–4^	476	161.3
Cu	*y* = −0.0002*x* + 2.6921	**0.980**	0.0005	492	*y* = 0.0013*x* + 3.5585	0.279	4.74 × 10^–7^	769	37.6
Zn	*y* = −7 × 10^–5^ *x* + 2.5534	0.959	0.0002	358	*y* = 0.0046*x* + 0.2596	**0.994**	2.60 × 10^–5^	385	3.9
As	*y* = −0.0004*x* + 2.3184	0.322	0.0009	208	*y* = 0.0034*x* – 0.0435	**0.999**	0.000266	294	23
Se	*y* = −1 × 10^–5^ + 2.2255	0.006	2 × 10^–5^	168	*y* = 0.003*x* – 0.0165	**1.000**	0.000545	333	60.6
Ag	*y* = −0.0007*x* + 2.3245	0.940	0.0016	211	*y* = 0.0023*x* + 0.077	**0.999**	6.87 × 10^–5^	435	13.0
Cd	*y* = −0.0009*x* + 2.0261	0.957	0.0021	106	*y* = 0.0021*x* + 0.0317	**1.000**	1.39 × 10^–4^	476	1.3
Ba	*y* = −0.0002*x* + 2.4529	0.965	0.0005	284	*y* = 0.0027*x* + 0.2138	**0.987**	3.41 × 10^–5^	370	4.7
Tl	*y* = −5 × 10^–5^ + 2.2875	0.164	0.0001	194	*y* = 0.0029*x* + 0.0516	**0.998**	0.000163	345	19.4
Pb	*y* = −5 × 10^–5^ + 2.6984	**0.984**	0.0001	499	*y* = 0.0037*x* + 16.097	0.033	8.5 × 10^–7^	270	0.06

a C: 1.0 mg/L; V: 100 mL, adsorbent: 0.2 g; *t*: 0, 15, 45, 75, 150, 270, 340, 1440 min.

**Table 5:** Parameters of Intraparticle Diffusion Kinetic Models^a^

	intraparticle diffusion-1				intraparticle diffusion-2			
	equation	*R* ^2^	*k*_int‑1_	I-1	equation	*R* ^2^	*k*_int‑2_	I-2
**Al**	*y* = 5.7237*x* + 30.077	0.934	5.7	30.1				
**Cr**	*y* = 1.9023*x* – 8.6199	0.925	1.9	–8.6				
**Mn**	*y* = 98.301*x* + 6 × 10^–14^	1.000	98.3	0.0	*y* = 2.0455*x* + 372.5	0.980	2.05	372.5
**Co**	*y* = 106.37*x* + 6 × 10^–14^	1.000	106.4	6 × 10^–14^	*y* = 1.2741*x* + 401.08	0.894	1.27	401.08
**Ni**	*y* = 0.4842*x* + 460.05	0.943	0.5	460.1				
**Cu**	*y* = 7.1732*x* – 29.039	0.959	7.2	–29.0				
**Zn**	*y* = 18.163*x* – 9.2267	0.895	18.2	–9.2	*y* = 2.4394*x* + 121.97	0.991	2.4	122.0
**As**	*y* = 38.711*x* + 31.656	0.924	38.7	31.7	*y* = −1.8942*x* + 369.24	0.945	–1.89	369.24
**Se**	*y* = 41.879*x* + 5.0871	0.989	41.9	5.1	*y* = 0.8207*x* + 360.84	0.944	0.8	360.8
**Ag**	*y* = 76.171*x*	1.000	76.2	0.0	*y* = 4.0175*x* + 283.44	0.914	4.0	282.4
**Cd**	*y* = 103.04*x* + 6 × 10^–14^	1.000	103.0	6 × 10^–14^	*y* = 2.4141*x* + 392.33	0.912	2.4	392.3
**Ba**	*y* = 25.229*x* + 12.235	1.000	25.2	12.2	*y* = 4.5278*x* + 180.46	0.918	4.5	180.5
**Tl**	*y* = 32.55*x*	1.000	32.6	0.0	*y* = 2.0175*x* + 264.76	0.953	2.0	264.8
**Pb**	*y* = 1.9861*x* – 9.4976	0.936	2.0	–9.5				

a C: 1.0 mg/L; V: 100 mL, adsorbent: 0.2 g; *t*: 0, 15, 45, 75, 150, 270, 340, 1440 min.

The variation in *k*_2_ values can be attributed to the sensitivity of the pseudo-second-order linearization to small deviations in slope, particularly when *q*_e_ values are similar. Therefore, model evaluation was based primarily on agreement between experimental and calculated *q*_e_ values and overall kinetic trends, while *R* ^2^ values were used only as supporting indicators.

Considering the results of the kinetic study, aluminum, chromium, copper and lead showed kinetic behavior closer to the pseudo-first-order model while other elements exhibited behavior more consistent with the pseudo-second order model. Similarly, representative nonlinear kinetic fitting analyses yielded trends consistent with the linearized models (Supporting Information, Figures S2 and S3), confirming that the linear models used in this study serve as reasonable comparative descriptors.

The intraparticle diffusion plots suggest a two-phase adsorption behavior, indicating that the process involves both surface adsorption and intraparticle diffusion. The intraparticle diffusion rate constants (*k*_int‑1_ and *k*_int‑2_) for each phase are shown in Table 5. The initial curved portion of the plots suggests that film diffusion may also contribute, particularly during the early stages, indicating that the adsorption is governed by a combination of film diffusion and intraparticle diffusion mechanisms. Additionally, the graphs for aluminum, chromium, nickel, copper and lead are linear but do not pass through the origin, meaning intraparticle diffusion alone is not the rate-determining step.

In the present study, the observed two-phase behavior in the intraparticle diffusion plots may also be related to the presence of the Fe_3_O_4_ coating on the biomass surface. The Fe_3_O_4_ layer can modify the surface morphology and pore characteristics, potentially increasing surface heterogeneity and creating additional active sites. While this modification enhances adsorption performance, it may also influence diffusion pathways within the porous structure, thereby contributing to the multistep adsorption mechanism observed in the kinetic analysis.

In addition to the kinetic observations, the adsorption mechanism of metal ions and metalloids onto the magnetite-coated biomass involves multiple types of interactions. For metal ions such as Pb^2+^, Cd^2+^, Cu^2+^, and Cr^3+^, coordination interactions are likely to occur with oxygen-containing functional groups (e.g., hydroxyl and carboxyl groups) on the biomass surface, and electrostatic interactions also contribute, particularly under conditions where the adsorbent surface is negatively charged. In contrast, metalloids such as As and Se may interact through surface complexation and ligand exchange mechanisms often forming inner-sphere complexes with surface hydroxyl groups. The presence of Fe_3_O_4_ nanoparticles enhances these interactions by providing additional active sites, increasing surface heterogeneity, and facilitating electron transfer, which can improve adsorption capacity for both metals and metalloids. This clarification explains why different adsorption mechanisms may dominate for metals versus metalloids under multielement conditions.

Furthermore, the selectivity of the developed adsorbent toward different metal ions has been evaluated. The adsorption experiments under multielement conditions revealed that certain metal ions (e.g., Pb^2+^, Cd^2+^, Cu^2+^) are preferentially adsorbed due to their higher affinity toward the functional groups on the adsorbent surface and favorable coordination interactions. In contrast, other ions exhibited lower adsorption, likely as a result of competition for active sites, differences in ionic radius, and hydration energy.

The kinetic and isotherm models were evaluated using linearized forms primarily for comparative assessment across multiple elements. Since linear transformations may alter error distribution and dependent variables differ among models, the selection of the best-fitting model was not based solely on *R* ^2^ values. Instead, agreement between experimental and calculated qe values and overall consistency of adsorption trends were considered. Therefore, the obtained parameters are used as comparative descriptors rather than definitive mechanistic indicators.

### 3.8 Applications to Real Water Samples – Validation Study

The adsorption method was also applied to real samples in order to check the accuracy of the method and analysis. Recovery (spiking) experiments were conducted to prove accuracy. For this purpose, drinking water and wastewater samples were analyzed using standard solutions. Recovery (%) values were calculated using the eq (eq 8) below. The results are shown in Table 6.

8 $$\mathrm{Recovery}\left(\%\right)=\frac{\left(S+\mathrm{Std}\right)-S}{\mathrm{Std}}\times 100$$

where *S* is the native sample concentration, Std is the added standard concentration, S + Std is the measured concentration after spiking, R is the recovery efficiency (%), ND is “Not Detected”; concentration below the detection limit of the analytical method. Removal efficiency was calculated considering the detection limit as the maximum possible residual concentration.

**Table 6:** Recovery Results for Drinking Water and Wastewater Samples^a^

	drinking water				waste water			
	*S* (μg/L)	Std (μg/L)	S+Std (μg/L)	R (%)	*S* (μg/L)	Std (μg/L)	S+Std (μg/L)	R (%)
Al	10.7 ± 1.1	194.7 ± 1.0	195.12 ± 0.81	**94.72 ± 0.67**	291 ± 13	248.9 ± 1.0	565.7 ± 4.6	**110 ± 24**
Cr	ND	200.4 ± 1.5	178.3 ± 2.5	**>88.93 ± 0.67**	0.470 ± 0.080	250.43 ± 0.60	218.5 ± 2.9	**87.06 ± 0.22**
Mn	0.320 ± 0.060	200.2 ± 1.3	196.5 ± 9.2	**97.99 ± 0.69**	14.0 ± 1.2	251.1 ± 1.1	254.1 ± 5.0	**95.6 ± 2.4**
Co	1.860 ± 0.030	195.1 ± 1.3	200.0 ± 2.6	**101.56 ± 0.68**	19.30 ± 0.50	244.87 ± 0.80	246.3 ± 2.7	**92.70 ± 0.63**
Ni	ND	199.7 ± 1.8	165.0 ± 2.3	**>82.48 ± 0.74**	1.000 ± 0.010	250.18 ± 0.70	210.8 ± 4.3	**83.86 ± 0.23**
Cu	1.350 ± 0.050	197.0 ± 2.0	181.0 ± 3.6	**91.19 ± 0.93**	9.4 ± 1.1	245.65 ± 0.90	227.4 ± 2.1	**88.74 ± 0.99**
Zn	22.30 ± 0.31	203.8 ± 2.3	264.5 ± 5.8	**118.8 ± 1.6**	337.1 ± 4.7	252.3 ± 1.1	559.8 ± 1.8	**88.3 ± 3.4**
As	0.100 ± 0.010	204.2 ± 1.8	179.8 ± 2.3	**>88.00 ± 0.78**	9.49 ± 0.80	250.90 ± 0.40	237.7 ± 2.3	**90.96 ± 0.75**
Se	ND	202.3 ± 1.7	222.4 ± 2.0	**>109.83 ± 0.92**	24.1 ± 2.7	251.4 ± 1.1	260.9 ± 3.0	**94.2 ± 3.2**
Ag	1.330 ± 0.040	203.9 ± 1.6	167.4 ± 4.5	**81.45 ± 0.65**	2.72 ± 0.11	255.0 ± 1.3	202.2 ± 1.5	**78.23 ± 0.40**
Cd	0.190 ± 0.020	198.95 ± 0.50	214.0 ± 3.2	**107.47 ± 0.27**	4.55 ± 0.15	249.30 ± 0.30	242.7 ± 1.1	**95.53 ± 0.13**
Ba	0.910 ± 0.080	198.8 ± 2.1	198 ± 12	**99.1 ± 1.2**	151.8 ± 3.6	250.65 ± 0.70	397.7 ± 4.3	**98.1 ± 6.2**
Tl	ND	199.8 ± 1.8	162.6 ± 2.3	**>81.38 ± 0.73**	4.280 ± 0.080	252.39 ± 0.90	230.9 ± 1.9	**89.79 ± 0.33**
Pb	2.340 ± 0.020	200.3 ± 2.1	179.4 ± 2.3	**88.40 ± 0.93**	15.88 ± 0.25	255.0 ± 1.1	222.6 ± 1.9	**81.07 ± 0.40**

a Mean ± SD, *n* = 3 for each element; total measurements = 84. ND: Not Detected.

Recoveries slightly exceeding 100% are attributed to matrix effects and instrumental uncertainty at trace concentration levels. Standard addition calibration was applied to compensate for matrix-induced signal enhancement or suppression. Each value represents the mean ± standard deviation of three independent replicates (*n* = 3).

It was seen that the recoveries were close to 100%. The accuracy of our data was confirmed by this experiment. The recovery value slightly above 100% obtained for Zn in the drinking water sample may be attributed to matrix-induced signal enhancement and minor analytical uncertainty, which is common in ICP-MS trace-level measurements. However, the value remains within the generally acceptable recovery range (80–120%) for environmental analyses.

The adsorption method was also applied to real water samples and certified standard materials. For this purpose, 4 different water samples were used. Twenty-five mL of solutions were added to 0.05 g of adsorbent and shaken for 24 h. The results are shown in Table 7.

**Table 7:** Application of the Magnetite-Coated Biomass Adsorbent to Real Water Samples^a^

	Sample 1			Sample 2			Sample 3			Sample 4		
	concentration (μg/L)			concentration (μg/L)			concentration (μg/L)			concentration (μg/L)		
	before	after	R. E. (%)	before	after	R. E. (%)	before	after	R. E. (%)	before	after	R. E. (%)
Al	237 ± 10	16.39 ± 0.60	**93.08** ± 0.57	195.12 ± 0.81	10.49 ± 0.70	**94.62** ± 0.38	291 ± 13	45.7 ± 6.2	**84.3** ± 2.8	565.7 ± 4.6	83.5 ± 5.3	**85.24** ± 0.84
Cr	207.5 ± 9.3	22.4 ± 0.80	**89.21** ± 0.92	178.3 ± 2.5	21.8 ± 1.1	**87.77** ± 0.85	0.470 ± 0.080	ND	**>86.0** ± 7.0	218.5 ± 2.9	20.6 ± 1.1	**90.57** ± 0.64
Mn	103.0 ± 8.0	1.10 ± 0.60	**98.93** ± 0.67	196.5 ± 9.2	1.8 ± 1.1	**99.08** ± 0.60	14.0 ± 1.2	1.36 ± 0.40	**90.3** ± 3.3	254.1 ± 5.0	6.8 ± 3.7	**97.3** ± 1.5
Co	1011 ± 12	1.90 ± 0.70	**99.81** ± 0.70	200.0 ± 2.6	2.9 ± 1.0	**98.55** ± 0.51	19.30 ± 0.50	0.24 ± 0.30	**98.8** ± 1.4	246.3 ± 2.7	3.5 ± 1.5	**98.58** ± 0.62
Ni	211 ± 11	2.93 ± 0.70	**98.61** ± 0.41	165.0 ± 2.3	ND	**>99.80** ± 0.03	1.000 ± 0.010	ND	**>72.0** ± 5.3	210.8 ± 4.3	1.21 ± 0.80	**99.43** ± 0.39
Cu	437.0 ± 8.9	25.40 ± 0.40	**94.19** ± 0.21	181.0 ± 3.6	16.7 ± 1.8	**90.8** ± 1.1	9.4 ± 1.1	1.80 ± 0.90	**81** ± 12	227.4 ± 2.1	8.2 ± 2.9	**96.4** ± 1.3
Zn	2775.8 ± 9.6	67.5 ± 3.1	**97.57** ± 0.12	264.5 ± 5.8	18.7 ± 1.3	**92.93** ± 0.65	337.1 ± 4.7	11.6 ± 4.7	**96.6** ± 1.4	559.8 ± 1.8	14.1 ± 1.0	**97.48** ± 0.18
As	96 ± 11	2.5 ± 1.0	**97.4** ± 1.4	179.8 ± 2.3	7.8 ± 1.6	**95.66** ± 0.90	9.49 ± 0.80	0.40 ± 0.40	**95.8** ± 4.6	237.7 ± 2.3	4.42 ± 0.20	**98.14** ± 0.11
Se	125 ± 14	3.00 ± 0.80	**97.60** ± 0.92	222.4 ± 2.0	14.99 ± 0.50	**93.26** ± 0.25	24.1 ± 2.7	2.3 ± 1.1	**90.5** ± 5.7	260.9 ± 3.0	9.00 ± 0.70	**96.55** ± 0.31
Ag	1.10 ± 0.40	ND	**>91.8** ± 4.5	167.4 ± 4.5	3.27 ± 0.80	**98.05** ± 0.52	2.72 ± 0.11	0.16 ± 0.20	**94.1** ± 7.1	202.2 ± 1.5	3.90 ± 0,90	**98.07** ± 0.45
Cd	238.1 ± 9.6	1.94 ± 0.30	**99.19** ± 0.16	214.0 ± 3.2	5.5 ± 1.4	**97.43** ± 0.66	4.55 ± 0.15	0.12 ± 0.20	**97.4** ± 3.9	242.7 ± 1.1	2.2 ± 0.20	**99.09** ± 0.10
Ba	621.4 ± 7.4	2.01 ± 1.30	**99.68** ± 0.21	198 ± 12	0.43 ± 0.20	**99.78** ± 0.12	151.8 ± 3.6	1.68 ± 0,90	**98.89** ± 0.59	397.7 ± 4.3	6.98 ± 0.30	**98.24** ± 0.10
Tl	72.7 ± 7.9	0.17 ± 0.10	**99.77** ± 0.16	162.6 ± 2.3	0.56 ± 0.40	**99.66** ± 0.24	4.280 ± 0.080	0.006 ± 0.010	**99.86** ± 0.20	230.9 ± 1.9	2.59 ± 0.20	**98.88** ± 0.11
Pb	472.9 ± 8.4	38.9 ± 1.1	**91.77** ± 0.34	179.4 ± 2.3	20.7 ± 5.4	**88.5** ± 3.0	15.88 ± 0.25	2.5 ± 1.7	**84** ± 11	222.6 ± 1.9	14.65 ± 0.30	**93.42** ± 0.19

a Removal efficiencies (R.E. %) were calculated based on the concentrations measured before and after adsorption. Values represent mean ± standard deviation (*n* = 3). ND: Not detected (below method detection limit).

Sample 1: certified drinking water

Sample 2: 200 μg/L metal solution spiked commercial drinking water

Sample 3: wastewater

Sample 4: 250 μg/L metal solution spiked wastewater

According to the results, the adsorption approach was successfully validated using real water samples, yielding consistently high removal efficiencies across pure water, drinking water, and wastewater matrices. These findings confirm that the prepared adsorbent maintains its performance under realistic water conditions rather than only in idealized laboratory systems. The recovery values obtained for the investigated elements were generally within the acceptable analytical range (80–120%) for environmental analyses. Minor deviations from 100% recovery may be attributed to matrix effects and trace-level analytical uncertainties commonly encountered in complex water samples.

Unlike many previous studies that primarily focus on single-metal removal under controlled conditions, this study demonstrates the applicability of a waste-derived, keratin-based biomass adsorbent for the simultaneous removal of multiple metal ions in complex aqueous environments. The results underscore the practical relevance of low-cost biomass-based adsorbents and highlight their potential for real-world water remediation applications.

### 3.9 Regeneration Study

The regeneration experiments were performed in a fixed-bed column system. Briefly, 2 g of adsorbent was packed into a glass column (1 cm inner diameter), and glass wool was placed at both the bottom and top of the column to hold the material in place. A 10 mL multielement solution at pH 7.0 (1000 μg/L each) was passed through the column using a peristaltic pump at a flow rate of 0.5 mL/min. Subsequently, 10 mL of regeneration solution (2% NaOH + 3% NaCl) was introduced at the same flow rate. This adsorption–desorption cycle was repeated four times.

Effective regeneration was observed only for Al, As, Sb and Se, whereas the remaining elements showed negligible desorption under the selected regeneration conditions. The adsorption–regeneration performance over successive cycles is presented in Figure 8.

**Figure 8:**
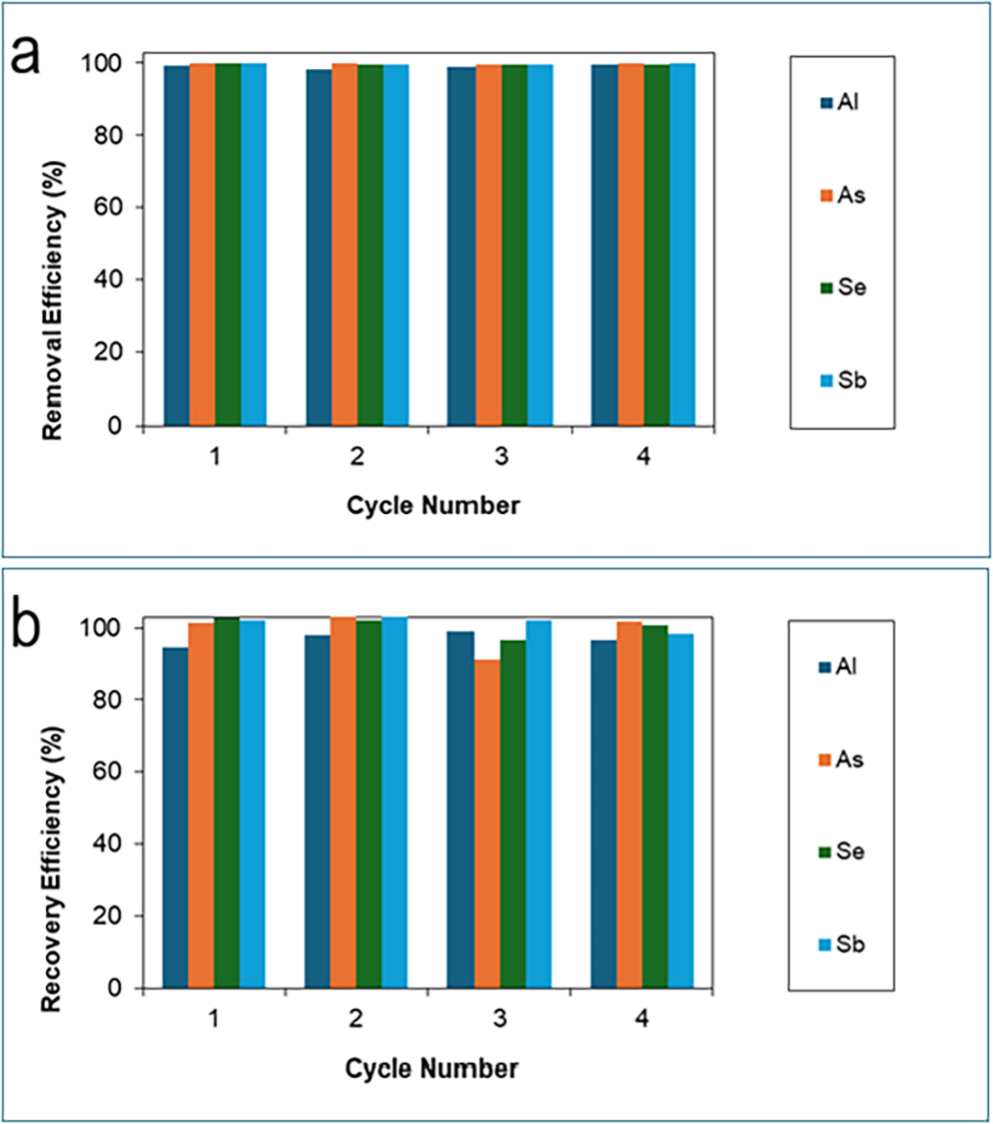
Regeneration performance of magnetite-coated biomass showing (a) adsorption and (b) desorption efficiencies of Al, As, Sb, and Se over four consecutive cycles in a fixed-bed column.

As shown in Figure 8, the adsorption capacity for Al, As, Sb, and Se remained relatively stable over repeated cycles, indicating that the adsorbent maintains its regeneration capability for these elements under the applied conditions.

Iron leaching during regeneration was also monitored using ICP-MS to evaluate the stability of the magnetite coating. In the first adsorption cycle, the Fe concentration in the treated solution was 62.6 ± 3.1 ppb. During the first regeneration step, the Fe concentration increased to 37.5 ± 3.7 μg/L, followed by 12.21 ± 0.56 μg/L in the second adsorption cycle and 5.030 ± 0.020 μg/L in the second regeneration step. In subsequent cycles, Fe concentrations remained approximately constant at ∼5 μg/L. The relatively higher Fe release observed in the first cycle is attributed to the removal of loosely bound magnetite particles and initial conditioning of the packed bed under alkaline regeneration conditions. The sharp decrease in Fe concentration in subsequent cycles and the stable values (∼5 μg/L) indicate that no progressive dissolution occurs and confirm the structural stability of the magnetite coating during repeated regeneration.

### 3.10 Comparison

A comparison of the keratin–Fe_3_O_4_ adsorbent developed in this study with recently reported magnetite-coated biomasses and other low-cost adsorbents (Table 8) demonstrates its notable multielement adsorption capability and reusability. While conventional magnetite-coated biomasses and modified biochars generally target one or a few metal ions (e.g., Cr^6+^, Pb^2+^, Cu^2+^, Zn^2+^, Cd^2+^), our keratin–Fe_3_O_4_ composite is capable of adsorbing 14 different metals and metalloids. The maximum adsorption capacity of the keratin–Fe_3_O_4_ composite ranges from 0.216 to 64.5 mg/g depending on the element, which is comparable to or higher than previously reported adsorbents, such as Fe-modified magnetic biochar (11.75–361.01 mg/g for Cr^6+^) and humic acid-modified Fe_3_O_4_ (33.3–111.1 mg/g for Pb^2+^, Cu^2+^, Cd^2+^, Ni^2+^).

**Table 8:** Comparison of Magnetite-Based Biomass Adsorbents

adsorbent	target species	isotherm model	max capacity (mg/g)	regeneration solution	refs
Fe-modified magnetic biochars	Cr^6+^	Langmuir	11.75 – 361.01		^34^
humic acid-modified Fe_3_O_4_	Pb^2+^, Cu^2+^, Cd^2+^, Ni^2+^	Langmuir	33.3 – 111.1	HCl	^35^
magnetic biochar (Pb^2+^ and Zn^2+^)	Pb^2+^, Zn^2+^	Langmuir	103.7 – 329.6	deionized water	^36^
magnetic chitosan/biochar composite	Cu^2+^	Langmuir	55.2		^37^
nanomagnetite biochar (As and F)	As, F		7.97 (As)		^38^
Fe_3_O_4_–teff straw biomass	Cd^2+^	Langmuir	101.0		^39^
magnetite impregnated lignocellulosic biomass	Zn^2+^	Freundlich	2.24 – 2.62		^40^
magnetite coated pine biomass	Cr^6+^	Langmuir	13.88		^41^
keratin-Fe_3_O_4_ biomass	14 metals/metalloids	Langmuir/Freundlich	0.216 – 64.5	2% NaOH + 3% NaCl	this study

Furthermore, unlike most reported adsorbents, our material can be regenerated using a 2% NaOH + 3% NaCl solution and reused effectively for several elements (Al, As, Sb, Se) without a significant loss in removal efficiency. This highlights the potential of the keratin–Fe_3_O_4_ composite for sustainable and cost-effective water treatment applications, particularly in multimetal systems, where many other adsorbents lack reusability or multielement coverage.

## 4 Discussion

In this study, a green adsorbent derived from waste biomass and coated with magnetite was successfully prepared for the simultaneous removal of multiple toxic metal ions from water. The material demonstrated effective adsorption performance toward 14 different elements, highlighting its potential for application in complex multielement aqueous systems.

Comprehensive characterization confirmed the formation of nanosized magnetite within the biomass matrix and a basic surface pH (pH_p_zc = 10.1), which significantly influences adsorption behavior. Adsorption capacity values ranged from 216 to 64461 μg/g, indicating strong and heterogeneous uptake across different metal species.

Kinetic and equilibrium studies revealed that adsorption behavior varies depending on the element type. The pseudo-second-order model generally provided a better description of the experimental data; however, model interpretation was revised to emphasize agreement between calculated and experimental q_e_ values and overall adsorption trends rather than relying solely on linear regression parameters. Representative nonlinear isotherm and kinetic analyses performed for selected elements (Supporting Information) showed trends consistent with the comparative linear analysis. The intraparticle diffusion analysis suggests that adsorption proceeds through multiple stages, involving both surface interaction and diffusion-controlled processes. This indicates that no single mechanism is solely responsible for the overall adsorption rate.

Real sample experiments showed satisfactory recoveries close to 100%, confirming the applicability of the method in complex environmental water matrices.

The adsorption behavior is governed by the combined contribution of keratin functional groups and Fe_3_O_4_ nanoparticles, enabling multiple interaction mechanisms including coordination, electrostatic attraction, and surface complexation.

Although the synthesis involves mild conditions and relatively low chemical demand, the term “green” is used in the context of low-cost preparation, waste valorization, and reduced process complexity compared to conventional magnetite synthesis routes.

Overall, the results demonstrate the potential of the developed composite as a promising multifunctional adsorbent for environmental remediation, while acknowledging that further refinement using nonlinear modeling and advanced spectroscopic validation would strengthen mechanistic understanding in future studies.

## Supplementary Material



## Notes

LOD and LOQ values of the elements (Table S1); calibration graphs of the elements (Figure S1); nonlinear Langmuir and Freundlich isotherm fitting for the adsorption of Cd and Co (Figure S2); and nonlinear pseudo-first-order (PFO) and pseudo-second-order (PSO) kinetic fitting for the adsorption of Cd and Co (Figure S3) (PDF)
