# Supplementary material for: A Green Magnetite-Coated Waste Biomass for Simultaneous Removal of Multiple Toxic Metals from Water
Source: ACS Omega. 2026 Jun 24;11(26):38594–606. doi: 10.1021/acsomega.6c00870 (PMC13347389; doi:10.1021/acsomega.6c00870)
Supplement: Supplementary file 1 [file ao6c00870_si_001.pdf]

Supporting Information

A green magnetite-coated waste biomass for simultaneous removal of multiple toxic metals from water

Tülin Deniz Çiftçi*[a], Yağmur Deniz Çiftçi [b], Ela Güngör [b], Zeliha Ada İplikçi [b], Serap Yıldırım Metin [b]

[a] Ege University, Faculty of Science, Department of Chemistry, Erzene, 35100, Bornova, İzmir, Türkiye

[b] İzmir Bahçeşehir College 50th Year Anatolian High School, Çiçekliköy, 35040, Bornova, İzmir, Türkiye

List of Supporting Information

- Table S1
- Figure S1
- Figure S2
- Figure S3

Table S1. LOD and LOQ values of the elements.

Element	LOD (µg/L)	LOQ (µg/L)
Al	1.96	6.53
Sb	0.02	0.07
As	0.07	0.23
Cu	0.36	1.20
Ba	0.25	0.83
Zn	0.31	1.03
Ag	0.09	0.30
Cd	0.02	0.07
Co	0.04	0.13
Cr	0.07	0.23
Pb	0.05	0.17
Mn	0.18	0.60
Ni	0.28	0.93
Se	0.21	0.70
Tl	0.01	0.03

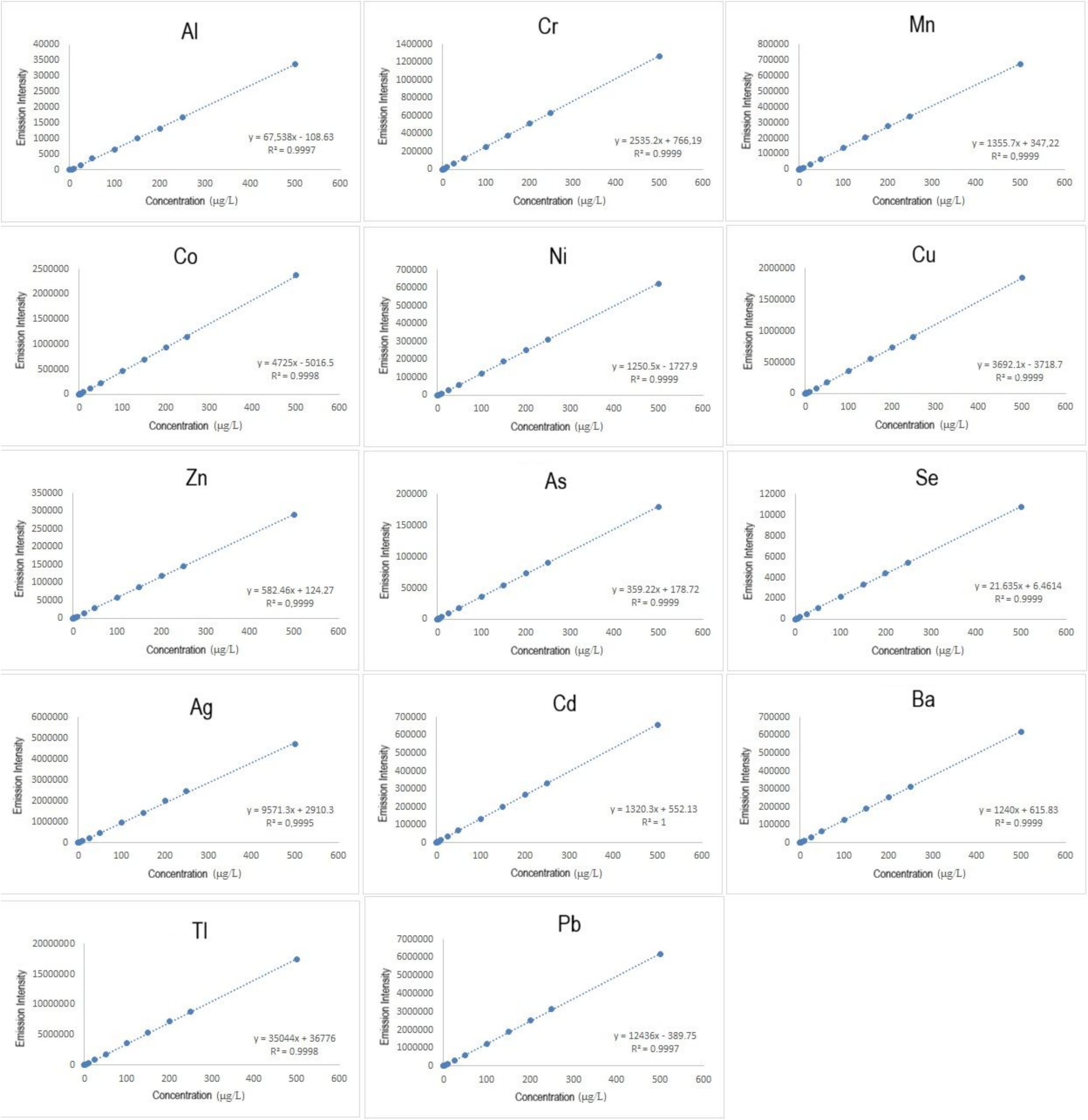

Figure S1. Calibration graphs of the elements a) Al, b) Cr, c) Mn, d) Co, e) Ni, f) Cu, g) Zn, h) As, i) Se, j) Ag, k) Cd, l) Ba, m) Tl, n) Pb.

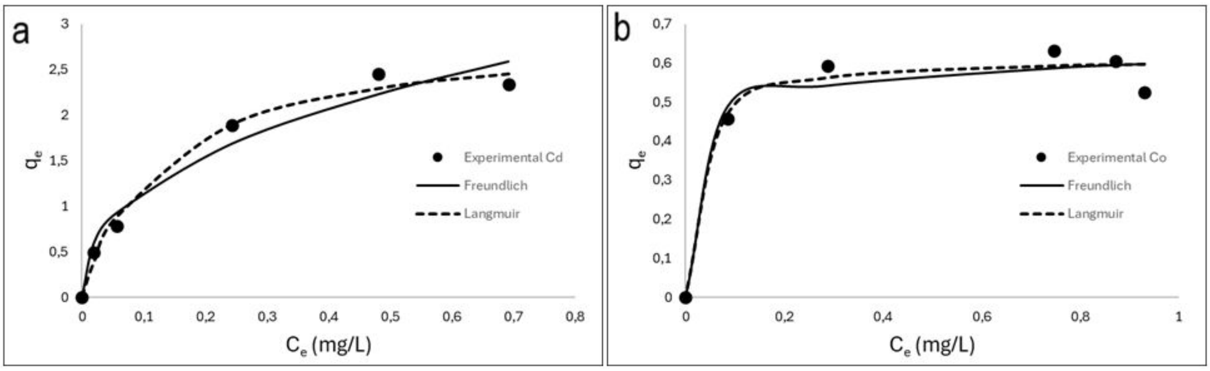

Figure S2. Nonlinear Langmuir and Freundlich isotherm fitting for the adsorption of a) Cd and b) Co.

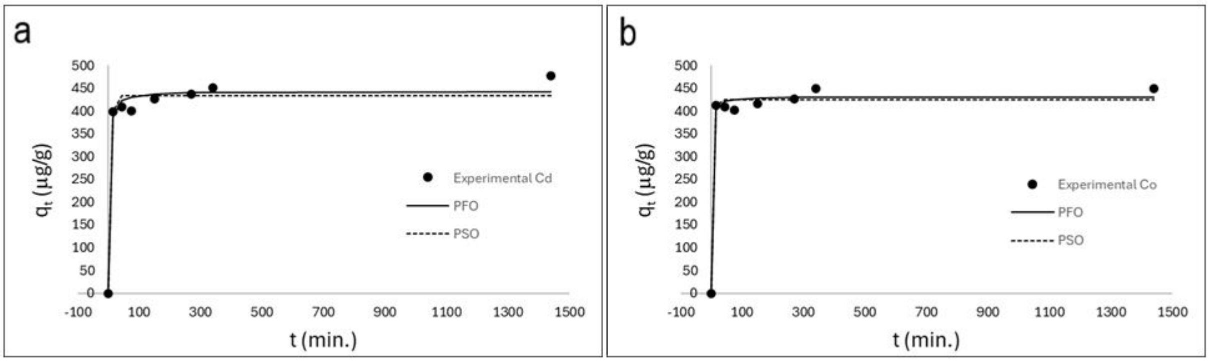

Figure S3. Nonlinear pseudo-first-order (PFO) and pseudo-second-order (PSO) kinetic fitting for the adsorption of a) Cd and b) Co.
